# *De Novo* Renal Cell Carcinoma in a Kidney Allograft 20 Years after Transplant

**DOI:** 10.1155/2015/679262

**Published:** 2015-02-19

**Authors:** Masataka Banshodani, Hideki Kawanishi, Seiji Marubayashi, Sadanori Shintaku, Misaki Moriishi, Fumio Shimamoto, Shinichiro Tsuchiya, Kiyohiko Dohi, Hideki Ohdan

**Affiliations:** ^1^Department of Artificial Organs, Akane-Foundation, Tsuchiya General Hospital, 3-30 Nakajimacho, Naka-ku, Hiroshima 730-8655, Japan; ^2^Department of Pathology, Faculty of Human Culture and Science, Prefectural University of Hiroshima, 1-1-71 Ujina-Higashi, Minami-ku, Hiroshima 734-8558, Japan; ^3^Department of Surgery, Akane-Foundation, Tsuchiya General Hospital, 3-30 Nakajimacho, Naka-ku, Hiroshima 730-8655, Japan; ^4^Department of Gastroenterological and Transplant Surgery, Applied Life Sciences, Institution of Biomedical & Health Sciences, Hiroshima University, 1-2-3 Kasumi, Minami-ku, Hiroshima 734-8551, Japan

## Abstract

Renal cell carcinoma (RCC) in a kidney allograft is rare. We report the successful diagnosis and treatment of a *de novo* RCC in a nonfunctioning kidney transplant 20 years after engraftment. A 54-year-old man received a kidney transplant from his mother when he was 34 years old. After 10 years, chronic rejection resulted in graft failure, and the patient became hemodialysis-dependent. Intravenous contrast-enhanced computed tomography (CT) for the evaluation of gastrointestinal symptoms revealed a solid 13 mm tumor in the kidney graft. The tumor was confirmed on ultrasound examination. This tumor had not been detected on a surveillance noncontrast CT scan. Needle biopsy showed that the tumor was an RCC. Allograft nephrectomy was performed. Pathological examination showed that the tumor was a Fuhrman Grade 2 RCC. XY-fluorescence hybridization analysis of the RCC showed that the tumor cells were of donor origin. One year after the surgery, the patient is alive and has no evidence of tumor recurrence. Regardless of whether a kidney transplant is functioning, it should periodically be imaged for RCC throughout the recipient's lifetime. In our experience, ultrasonography or CT with intravenous contrast is better than CT without contrast for the detection of tumor in a nonfunctioning kidney transplant.

## 1. Introduction

Renal cell carcinoma (RCC) is more likely to occur in a native kidney of a transplant recipient than in the general population [1]. On the other hand, RCC in a kidney allograft is rare [2]. In a previous study, kidney carcinomas represented 4.6% of posttransplant cancers, as compared with 3% of tumors in the general population. However, among these carcinomas, only 10% occurred in kidney grafts [3]. Therefore, the management of RCC in a kidney graft has not yet been established. Kidney graft tumors can be carcinomas that are transmitted by donors, metastatic carcinomas from the recipient's native organs, or* de novo* carcinomas that occur after transplant. Identification of the origin of a kidney graft tumor can improve therapeutic safety and certainty. Here, we report the successful diagnosis and treatment of a* de novo* RCC in a nonfunctioning kidney transplant 20 years after engraftment.

## 2. Case Presentation

**Figure 1:**
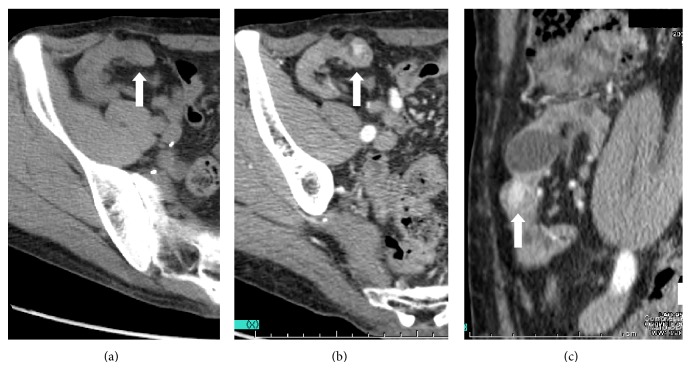
Computed tomography images. (a) A noncontrast computed tomography (CT) scan in the horizontal view, revealing no tumor in the kidney graft. (b) and (c) Intravenous contrast-enhanced CT scans in horizontal (b) and sagittal (c) views showing an enhanced solid tumor in the kidney graft (*white arrows*).

**Figure 2:**
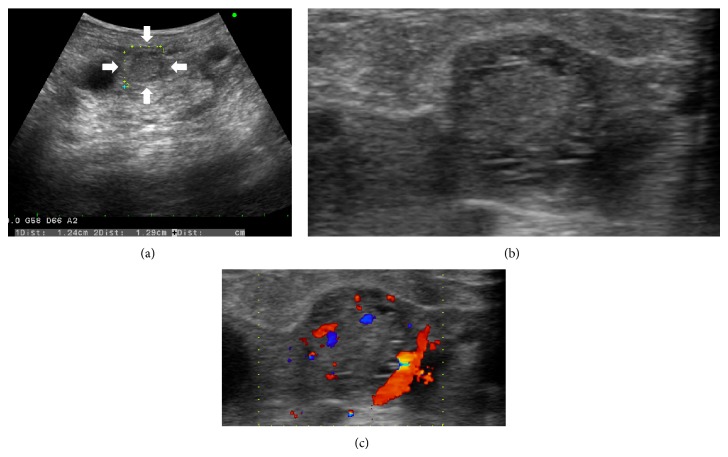
Ultrasonography images. (a) and (b) Ultrasonography revealing a solid tumor with a hypoechoic rim in the superficial cortex of the kidney graft (*white arrows*). (c) The hypervascular nature of the kidney graft tumor is confirmed on a Doppler image.

**Figure 3:**
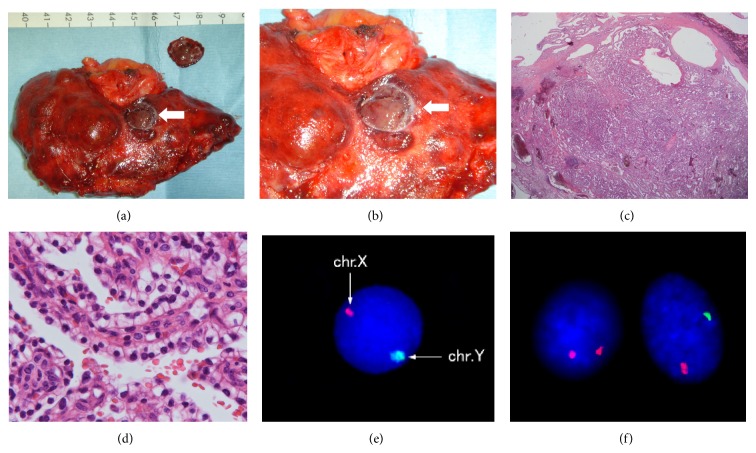
Pathological tissue images. (a) and (b) Allograft nephrectomy revealed that the tumor was 13 mm, Grade G2, INFb, v0, ly0, eg, fc1, im0, rc-inf1, rp-inf0, s-inf0, pT1a, pN0, pM0, and stage I. (c) and (d) On pathological examination (hematoxylin and eosin staining), the tumor was composed almost exclusively of clear cells that exhibited trabecular or papillary growth patterns in (c) low-power (10x) and (d) high-power (400x) fields. (e) and (f) An XY-fluorescence* in situ* hybridization (FISH) analysis performed on the kidney graft tumor revealing (e) healthy XX genotype control cells (lymphocytes) as well as (f) XX genotype cells (*left*) and XY genotype cells (*right*) in the tumor, including blood cells from the recipient. chr., chromosome.

A 54-year-old man with a history of immunoglobulin A nephropathy received a sex-mismatched kidney transplant (left kidney graft) from his mother when he was 34 years old. The donor underwent angiographic examination to ensure that there is no tumor stain in the left kidney (i.e., she had not developed a kidney tumor) prior to the transplant. Immunosuppression was induced with cyclosporine, methylprednisolone, mizoribine, and antilymphocyte globulin treatment for 20 days and was maintained with cyclosporine, methylprednisolone, and mizoribine treatment. After 10 years, chronic rejection led to kidney graft failure, and the patient became hemodialysis-dependent. After the transplant, he underwent a noncontrast computed tomography (CT) examination every year, but no tumor became evident in the kidney graft. Twenty years after engraftment, intravenous contrast-enhanced CT for the evaluation of gastrointestinal symptoms revealed an enhanced solid tumor with a diameter of approximately 13 mm in the kidney graft (Figures 1(b) and 1(c)). This tumor was not seen on noncontrast CT performed at the same time (Figure 1(a)). No other abnormal tumor shadows were observed. Ultrasonography revealed a solid 12.9 mm tumor with a hypoechoic rim in the superficial cortex of the kidney graft (Figures 2(a) and 2(b)). The hypervascular nature of the tumor was confirmed on Doppler imaging (Figure 2(c)). Allograft nephrectomy was performed after RCC was identified in a needle biopsy specimen of the graft tumor. Macroscopically, the tumor was a solid nodule, 13 mm wide, and encapsulated with a clear margin (Figures 3(a) and 3(b)). On pathological examination, the tumor was composed almost exclusively of clear cells that exhibited trabecular or papillary growth patterns, and the cells partially involved the capsule surrounding the tumor (Figures 3(c) and 3(d)). The tumor was diagnosed as Grade 2 clear cell carcinoma, according to the Fuhrman classification. XY-fluorescence* in situ* hybridization (XY-FISH) analysis was performed on the graft tumor; an XX genotype was seen in 85% of the tumor cells and an XY genotype was seen in 15% of the tumor cells, including the blood cells from the recipient (Figures 3(e) and 3(f)). These findings showed that the tumor originated from the donor tissue. The patient recovered uneventfully and was discharged 11 days after the operation. One year after the surgery, he is alive and has no evidence of tumor recurrence.

## 3. Discussion

Cases of RCCs in kidney allografts have rarely been reported. The incidence of* de novo* carcinomas in kidney allografts is reported to be 0.19–0.5% [2, 4]. Periodic ultrasonography and CT are useful for diagnosing kidney graft tumors [5, 6]. The time from kidney transplant to detection of a tumor in the kidney graft has ranged from 9 months to 29 years [4, 6, 7]. In our case, 20 years after engraftment, a tumor was detected in the tumor graft on intravenous contrast-enhanced CT and ultrasonography; however, it was not visible on noncontrast CT.

Kidney graft tumors can be carcinomas that are transmitted by donors, metastatic carcinomas from native recipient organs, or* de novo* carcinomas that occur after transplant. When a kidney graft tumor is detected within a short period after kidney transplant, the tumor is likely transmitted by the donor. However, the growth rate of kidney graft tumors is unknown. One study reported that* de novo* kidney graft carcinoma rapidly developed 9 months after transplant [8]. On the other hand, Tillou et al. concluded that* de novo* kidney graft tumors are predominantly low grade and not aggressive [2]. Therefore, it is difficult to accurately identify the tumor's origin based on the period between kidney transplant and tumor detection. Lotan and Laufer [9] identified the origin of a kidney graft tumor by performing DNA analysis. Moreover, another study reported that XY-FISH was useful for identifying the tumor's origin in cases of sex-mismatched kidney transplants [10]. Overall, however, the tumor's origin has been identified in few cases. In our case, the tumor's origin was successfully identified by XY-FISH analysis.

Kidney graft tumors are usually treated surgically. Allograft nephrectomy is justified when a tumor is >40 mm in size, deeply located within the graft, or a recurrence. On the other hand, nephron-sparing surgery can maintain quality of life by retaining kidney graft function and is therefore justified for small tumors with low risks of recurrence [2, 4]. In our case, the kidney graft tumor was treated with allograft nephrectomy because the graft was nonfunctional.

In an analysis of histological types, Tillou et al. [2] reported that papillary carcinomas represented >50% of kidney graft tumors and that low-grade tumors (Fuhrman Grades 1 and 2) accounted for 65% of kidney graft tumors. In our case, the tumor was diagnosed as a Fuhrman Grade 2 clear cell carcinoma.

In the present case, a tumor in a nonfunctional kidney graft was successfully treated with allograft nephrectomy. Further, the tumor's origin was successfully identified by XY-FISH analysis, 20 years after engraftment. To detect RCC, a kidney transplant should be imaged periodically throughout a recipient's lifetime, regardless of whether the kidney transplant is functioning. In our experience, ultrasonography or CT with intravenous contrast is better than CT without contrast for the detection of a tumor in a nonfunctioning kidney transplant.
